# Sexual conflict in twins: male co-twins reduce fitness of female Soay sheep

**DOI:** 10.1098/rsbl.2009.0366

**Published:** 2009-06-03

**Authors:** Peter Korsten, Tim Clutton-Brock, Jill G. Pilkington, Josephine M. Pemberton, Loeske E. B. Kruuk

**Affiliations:** 1 Institute of Evolutionary Biology, School of Biological Sciences, University of Edinburgh, West Mains Road, Edinburgh EH9 3JT, UK; 2 Department of Zoology, University of Cambridge, Downing Street, Cambridge CB2 3EJ, UK

**Keywords:** twinning, sexual conflict, sibling competition, reproductive success, prenatal hormones, masculinization

## Abstract

Males and females often have different requirements during early development, leading to sex-specific interactions between developing offspring. In polytocous mammals, competition for limited resources *in utero* may be asymmetrical between the sexes, and androgens produced by male foetuses could have adverse effects on the development of females, with potentially long-lasting consequences. We show here, in an unmanaged population of Soay sheep, that female lambs with a male co-twin have reduced birth weight relative to those with a female co-twin, while there was no such effect in male twins. In addition, females with a male co-twin had lower lifetime breeding success, which appeared to be mainly driven by differences in first-year survival. These results show that sex-specific sibling interactions can have long-term consequences for survival and reproduction, with potentially important implications for optimal sex allocation.

## 1. Introduction

Males and females often show large differences in morphology, physiology and behaviour, leading to different requirements during their early development (Clutton-Brock 1991). As a result, sex-specific interactions between developing offspring can arise, with potentially long-term fitness consequences (Uller 2006). In polytocous mammals, there may be asymmetries between the sexes in the demand and competition for resources *in utero*, particularly in sexually dimorphic species (Beatty 1956), and androgens produced by male foetuses could negatively affect the development of females (Ryan & Vandenbergh 2002). In this paper, we simultaneously investigate the short- and long-term effects of sex-specific interactions between twin siblings during early development.

Evidence suggests that asymmetrical resource competition between males and females in mixed-sex litters can have consequences for birth weight in a variety of mammal species (e.g. humans: James 2002; domestic sheep: Beatty 1956; Burfening 1972; but see Avdi & Driancourt 1997; and Saiga antelope: Kühl *et al*. 2007). However, despite strong evidence that pre-natal resource availability and birth weight can both have substantial effects throughout an individual's life (Lummaa & Clutton-Brock 2002), we know little about the long-term implications of *in utero* intersexual resource competition.

In addition to having different energy requirements, developing male foetuses require and produce androgens, in particular testosterone, in higher concentrations than developing females. These androgens leak through the foetal membranes, leading to increased androgen exposure of females positioned close to males. Thus in rodents, the presence of neighbouring male foetuses *in utero* can lead to long-lasting negative effects on females, such as delayed maturation, reduced sexual attractiveness and a shorter reproductive lifespan. Similarly, male foetuses may be negatively affected by neighbouring females (Ryan & Vandenbergh 2002). Prenatal hormonal interactions between offspring of different sexes are well described in rodents, but less is known about such effects in non-model systems.

Recently, Lummaa *et al*. (2007) presented data on pre-industrial human twins, showing that females with a twin brother had reduced lifetime reproductive success compared with females with a twin sister, as a result of a lower probability of marrying as well as decreased fecundity. They hypothesized that these effects were the consequence of females with a male co-twin being masculinized by increased prenatal androgen exposure, although it seems conceivable that long-term effects of differential allocation of pre-natal resources could also have contributed to the differences. The result was not replicated in three contemporary human populations (Medland *et al*. 2008).

Here we use data from a long-term study on an unmanaged population of Soay sheep to test the effect of co-twin sex (i) on male and female birth weight and (ii) on female lifetime breeding success (LBS).

## 2. Material and methods

### (a) Study system

Data were collected in a long-term study on an unmanaged population of Soay sheep (*Ovis aries*) on the island of Hirta within the St Kilda archipelago, Scotland. Since 1985, individuals in the Village Bay study population have been intensively monitored throughout their lives. Approximately 95 per cent of the lambs are captured for ear tagging and weighing within a few days of birth. Approximately 15 per cent of births are twins, while the other 85 per cent consists of singletons (Clutton-Brock & Pemberton 2004); among twins, only 26 per cent are full siblings, whereas the remainder have different fathers (Pemberton *et al*. 1999). Adults are weighed during annual round-ups in August, in which approximately 65 per cent of individuals of the study population are captured each time. For details, see Clutton-Brock & Pemberton (2004). We used data from individuals born from 1986 to 2007. Only twins of which the sex of both lambs was recorded were included in the analyses (*n* = 379 twins, of which subsets of individuals were used for the analyses of birth weight and LBS).

### (b) Analysis of birth weight

We only included lambs' weights (hereafter, birth weight) if lambs were captured within 7 days after birth (of these, 89.0% were captured within 3 days). We used linear mixed effect models to test the effects of an individual's sex and its co-twin sex on its birth weight. We controlled for variation in capture age by including it (in days) as a covariate. We included the interaction terms of sex and co-twin sex to test for sex-specific interactions between siblings. After Lindström *et al*. (2002), we additionally included the following covariates: birth date (since 1 January; centred) and the mother's age and weight in the preceding August (both centred; linear and quadratic effects). To control for variation in environmental quality during gestation, we included the population densities in the year of the lamb's birth (*n*_*t*_) and the year before (*n*_*t*−1_) (Lindström *et al*. 2002). Altogether, 354 lambs of 184 litters born to 119 mothers were included. We included the identities (IDs) of mothers and litters as random effects to account for non-independence of lambs from the same mothers or litters. We also included birth year as a random effect.

### (c) Analysis of female reproductive success

LBS was estimated as the total number of lambs born to a female over her entire life; i.e. only females recorded to be dead were included (mean ± SD = 0.90 ± 2.57 lambs). We used Poisson-lognormal generalized linear mixed models with log link function to estimate the effect of co-twin sex on female LBS. Birth date and residuals of the regression of birth weight on capture age were included as fixed effects. Birth year was included as a random effect. Lamb ID was included as an additional random effect, to account for overdispersion in the data over and above the variance modelled by the fixed effects and by the Poisson random variation around the annual means (Elston *et al*. 2001). Altogether, 305 lambs of 233 litters born to 145 mothers were included. LBS data were only available for one of the two twins in 69.1 per cent of litters and for only one offspring per mother for 44.1 per cent of mothers, so it was not possible to also include litter and mother IDs as random effects. To investigate whether differences in LBS were driven by variation in first-year survival, we added first-year survival of lambs (until 1 May the following year; Wilson *et al*. 2005) to the final model.

**Figure 1.**
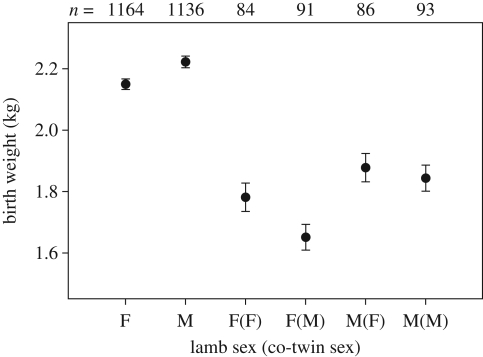
Mean (±s.e.) birth weight of Soay sheep lambs in relation to the composition of the litter in which they were born.

**Figure 2.**
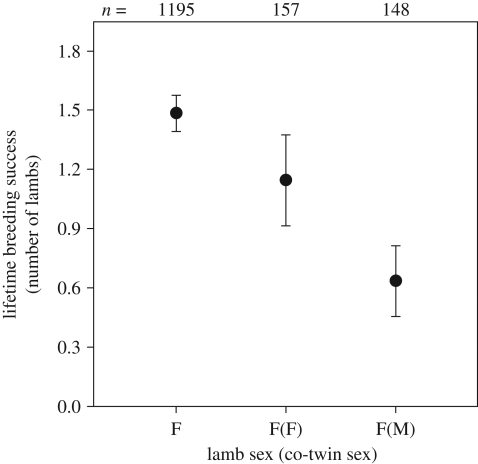
Mean (±s.e.) LBS of Soay sheep twin females in relation to the sex of their co-twin.

Because we are interested in the effect of co-twin sex on birth weight and LBS, we restrict statistical analyses to data from twins; however, for comparison, values for singletons are shown in figures 1 and 2. Models were implemented in GenStat 10.2.

## 3. Results

### (a) Effects of co-twin sex on birth weight

After controlling for the various variables known to explain variation in birth weight (Lindström *et al*. 2002), the interaction of lamb sex × co-twin sex had a significant effect on birth weight (table 1). This significant interaction was owing to a 10.0 per cent (0.18 kg) reduction in birth weight (predicted means) of female lambs with a male co-twin relative to those with a female co-twin, while the weight of males only differed 1.4 per cent (0.03 kg), depending on the sex of their co-twin (figure 1).

**Table 1.** Summary of the general linear mixed model fitting birth weight of Soay sheep twin lambs in relation to their own sex, the sex of their co-twin and the interaction of their own sex and co-twin sex, as well as other variables known to explain variation in birth weight (see §2). (Significance of model terms was assessed by adding them sequentially to produce the final model. *Lamb sex* gives weight of male versus female lambs; *co-twin sex* gives weight of lambs with a male versus female co-twin; *n*_*t*_ is the population density in the year of the lamb's birth; *n*_*t*−1_ is the density in the previous year (*n* = 354 lambs of 184 litters of 119 mothers).)

fixed effects	parameter estimate (s.e.)	*F*-value	d.f.	*p*-value
included				
constant	1.826 (0.146)			
capture age (in days)	0.105 (0.014)	43.02	1,227.4	<0.001
birth date	0.0156 (0.0032)	26.67	1,155.3	<0.001
*n*_*t*_	0.0011 (0.0002)	12.29	1,18.5	0.002
*n*_*t*−1_	−0.0017 (0.0002)	56.52	1,20.5	<0.001
mother's age	0.0129 (0.010)	5.02	1,158.9	0.026
mother's age^2^	−0.0094 (0.0033)	14.42	1,148.9	<0.001
mother's weight (kg)	0.0378 (0.0087)	17.85	1,172.1	<0.001
lamb sex	0.0231 (0.051)	26.34	1,271.5	<0.001
co-twin sex	−0.180 (0.050)	5.19	1,268.0	0.024
lamb sex × co-twin sex	0.206 (0.074)	7.66	1,152.9	0.006
excluded				
mother's weight^2^		0.18	1,175.5	0.67

variance components	*Ω* (s.e.)	

birth year	0.0047 (0.0041)				
mother ID	0.028 (0.0095)				
litter ID	0.010 (0.0082)			
residual	0.061 (0.0066)			

### (b) Effects of co-twin sex on female lifetime breeding success

Females with a male co-twin had significantly reduced LBS compared with females with a female co-twin (table 2 and figure 2). This reduction was significant after taking into account birth weight, which had a significant positive effect on LBS (table 2). The difference in LBS between females with male versus female co-twins appears to be largely driven by differences in first-year survival. When added to the model, first-year survival explained significant variation in LBS (Wald = 77.34, d.f. = 1, *p* < 0.001; see electronic supplementary material, table S1), and after taking account of this effect, the effect of co-twin sex was no longer significant (Wald = 2.88, d.f. = 1, *p* = 0.090).

**Table 2.** Summary of the Poisson-lognormal generalized linear mixed model fitting lifetime reproductive success of twin female Soay sheep in relation to the sex of their co-twin while controlling for effects of population density (*n*_*t*_, *n*_*t*−1_) and birth weight (residuals controlled for capture age). Wald values are sequential (*n* = 305 lambs; for details see §2 and table 1).

fixed effects	parameter estimate (s.e.)	Wald	d.f.	*p*-value
included				
constant	6.506 (1.213)			
*n*_*t*_	−0.0167 (0.0027)	31.77	1	<0.001
*n*_*t*−1_	−0.0020 (0.0019)	6.93	1	0.008
residual birth weight	1.301 (0.481)	8.41	1	0.004
co-twin sex	−0.806 (0.324)	6.19	1	0.013
excluded				
birth date		0.10	1	0.75

variance components	*Ω* (s.e.)	

birth year	0.224 (0.268)				
lamb ID	1.979 (0.392)				

## 4. Discussion

It has previously been shown in Soay sheep that having a twin sibling substantially reduces birth weight, leading to subsequent lower first-year survival, which ultimately results in reduced lifetime reproductive success (Wilson *et al*. 2005; see also figures 1 and 2). Here we show that there is additionally a sex-specific effect of having a twin sibling: female birth weight is more reduced by the presence of a male co-twin, than by a female co-twin, while for males, the reduction is not related to their co-twin's sex. These prenatal sex-specific interactions may also have long-term consequences, as females with a male co-twin had lower LBS compared with those with a female co-twin, even when controlling for the differences in birth weight. Continuing sex-specific competition over maternal resources (e.g. milk) after birth may contribute further to the differences in LBS of females in relation to their co-twin's sex. This seems not unlikely, given also that the effect on LBS was probably largely driven by differences in first-year survival. Similar effects of co-twin sex on birth weight and survival have been found in domestic sheep (Beatty 1956; Burfening 1972; but see Avdi & Driancourt 1997).

The observed sex-specific effects on birth weight and LBS may fit a pattern of hormonal interference between foetuses of different sexes (Ryan & Vandenbergh 2002; Lummaa *et al*. 2007). However, the observed reduction in female size does not necessarily fit with a hypothesis of ‘masculinization’ of female phenotypes, and it therefore seems more likely that competition over limited resources *in utero* importantly contributes to the observed pattern. Male Soay sheep lambs are on average heavier at birth than females (Lindström *et al*. 2002; see also figure 1) and may thus demand more resources during prenatal development, which could negatively affect female co-twins. Apparently, the competition is asymmetric between the sexes, as developing males are not affected by the presence of a male co-twin.

Remarkably, Kühl *et al*. (2007) recently found an opposite pattern in the highly sexually dimorphic Saiga antelope: males with a female co-twin had reduced birth weight compared with males with a male co-twin, while in females, there was no effect of co-twin sex. Kühl *et al*. speculated that perhaps the cumulative hormone secretion of male–male twins is necessary to induce the higher maternal investment needed to sufficiently provision twin males *in utero*. Another possibility is that females bearing male–male twins are on average in better condition and, as a result, invest more in their offspring *in utero*. Nevertheless, in this system, as in the Soay sheep, mixed-sex twins seem to have reduced fitness, and hence selection may favour females that overproduce same-sex twins (Uller 2006). In Soay sheep, however, we find no evidence for a bias towards same-sex twins (frequencies of twins, MM : MF : FF = 87 : 193 : 99; *χ*^2^ = 0.889, d.f. = 2, *p* = 0.64).

## References

[RSBL20090366C1] Avdi M. Driancourt M. A. 1997 Influence of sex ratio during multiple pregnancies on productive and reproductive parameters of lambs and ewes. Reprod. Nutr. Dev. 37, 21–27 (doi:10.1051/rnd:19970103) 9115592 10.1051/rnd:19970103

[RSBL20090366C2] Beatty R. A. 1956 Relation between genetic constitution of an offspring and weight of its litter-mates. Nature 178, 48–49 (doi:10.1038/178048b0) 13334532 10.1038/178048b0

[RSBL20090366C3] Burfening P. J. 1972 Prenatal and postnatal competition among twin lambs. Anim. Prod. 15, 61–66

[RSBL20090366C4] Clutton-Brock T. H. 1991 The evolution of parental care Princeton, NJ: Princeton University Press

[RSBL20090366C5] Clutton-Brock T. H. Pemberton J. M. 2004 Soay sheep: dynamics and selection in an island population Cambridge, UK: Cambridge University Press

[RSBL20090366C6] Elston D. A. Moss R. Boulinier T. Arrowsmith C. Lambin X. 2001 Analysis of aggregation, a worked example: numbers of ticks on red grouse chicks. Parasitology 122, 563–569 (doi:10.1017/S0031182001007740) 11393830 10.1017/s0031182001007740

[RSBL20090366C7] James W. H. 2002 Birth weight in dizygotic twins. Twin Res. 5, 309 12236189 10.1375/twin.5.4.309

[RSBL20090366C8] Kühl A. Mysterud A. Erdnenov G. I. Lushchekina A. A. Grachev I. A. Bekenov A. B. Milner-Gulland E. J. 2007 The ‘big spenders’ of the steppe: sex-specific maternal allocation and twinning in the Saiga antelope. Proc. R. Soc. B 274, 1293–1299 (doi:10.1098/rspb.2007.0038) 10.1098/rspb.2007.0038 PMC2176182 17341456

[RSBL20090366C9] Lindström J. Coulson T. Kruuk L. E. B. Forchhammer M. C. Coltman D. W. Clutton-Brock T. 2002 Sex-ratio variation in Soay sheep. Behav. Ecol. Sociobiol. 53, 25–30 (doi:10.1007/s00265-002-0545-4)

[RSBL20090366C10] Lummaa V. Clutton-Brock T. 2002 Early development, survival and reproduction in humans. Trends Ecol. Evol. 17, 141–147 (doi:10.1016/S0169-5347(01)02414-4)

[RSBL20090366C11] Lummaa V. Pettay J. E. Russell A. F. 2007 Male twins reduce fitness of female co-twins in humans. Proc. Natl Acad. Sci. USA 104, 10 915–10 920 (doi:10.1073/pnas.0605875104) 10.1073/pnas.0605875104 PMC1904168 17576931

[RSBL20090366C12] Medland S. E. Loehlin J. C. Willemsen G. Hatemi P. K. Keller M. C. Boomsma D. I. Eaves L. J. Martin N. G. 2008 Males do not reduce the fitness of their female co-twins in contemporary samples. Twin Res. Hum. Genet. 11, 481–487 (doi:10.1375/twin.11.5.481) 18828730 10.1375/twin.11.5.481 PMC4041993

[RSBL20090366C13] Pemberton J. M. Coltman D. W. Smith J. A. Pilkington J. G. 1999 Molecular analysis of a promiscuous, fluctuating mating system. Biol. J. Linn. Soc. 68, 289–301 (doi:10.1111/j.1095-8312.1999.tb01170.x)

[RSBL20090366C14] Ryan B. C. Vandenbergh J. G. 2002 Intrauterine position effects. Neurosci. Biobehav. Rev. 26, 665–678 (doi:10.1016/S0149-7634(02)00038-6) 12479841 10.1016/s0149-7634(02)00038-6

[RSBL20090366C15] Uller T. 2006 Sex-specific sibling interactions and offspring fitness in vertebrates: patterns and implications for maternal sex ratios. Biol. Rev. 81, 207–217 (doi:10.1017/S1464793105006962) 16677432 10.1017/S1464793105006962

[RSBL20090366C16] Wilson A. J. Pilkington J. G. Pemberton J. M. Coltman D. W. Overall A. D. J. Byrne K. A. Kruuk L. E. B. 2005 Selection on mothers and offspring: whose phenotype is it and does it matter? Evolution 59, 451–463 15807429

